# Randomized trial of acupressure to improve patient satisfaction and quality of recovery in hospitalized patients: study protocol for a randomized controlled trial

**DOI:** 10.1186/s13063-017-1839-1

**Published:** 2017-03-07

**Authors:** Eric Noll, Shivam Shodhan, Maria Cecilia Madariaga, Christopher R. Page, Diane Santangelo, Xiaojun Guo, Ehab Al Bizri, Aurora D. Pryor, Jamie Romeiser, Elliott Bennett-Guerrero

**Affiliations:** 1 Department of Anesthesiology Stony Brook Medicine, Stony Brook, NY USA; 2 Institut de Chirurgie Guidee par l’Image, IHU Hopitaux Universitaire de Strasbourg, Strasbourg, France; 3 Service d’Anesthesie Reanimation Hautepierre - CCOM - CMCO, Hopitaux Universitaires de Strasbourg, Strasbourg, France; 4 Department of Surgery Stony Brook Medicine, Stony Brook, NY USA; 5 C.C.O.M., 10 avenue Achille Baumann, 67400 Illkirch-Graffenstaden, France

**Keywords:** Quality of recovery, Acupressure therapy, Patient satisfaction

## Abstract

**Background:**

Acupressure therapy may be potentially beneficial in improving postoperative symptoms like postoperative nausea and vomiting (PONV), pain and sleep disorder and improving postoperative quality of recovery. The primary aim of this study is to investigate the efficacy of acupressure therapy on postoperative patient satisfaction and quality of recovery in hospitalized patients after surgical treatment.

**Methods/design:**

This three-group, parallel, superiority, blinded, randomized controlled trial will test the hypothesis that a combination of PC6, LI4 and HT7 acupressure is superior to sham or no intervention for improving postoperative quality of recovery in hospitalized patients. A minimum of 150 patients will be randomly allocated to one of the three experimental groups: control (no visit), light touch (sham acupressure) or active acupressure therapy in a 1:1:1 ratio. Interventions will be performed three times a day for 2 days. Patient satisfaction, quality of recovery, PONV and pain will be measured during the 3 days following randomization. The study protocol was approved by the Stony Brook University Institutional Review Board on 21 March 2016. Written informed consent will be recorded from every consented patient.

**Discussion:**

This study has the potential to improve the recovery of hospitalized patients by adding knowledge on the efficacy of acupressure therapy in this setting. A multipoint acupressure protocol will be compared to both a no intervention group and a light touch group, providing insight into different aspects of the placebo effect.

**Trial registration:**

ClinicalTrial.gov, NCT02762435. Registered on 14 April 2016.

**Electronic supplementary material:**

The online version of this article (doi:10.1186/s13063-017-1839-1) contains supplementary material, which is available to authorized users.

## Background

More than 200 million major surgical procedures are performed worldwide each year [1], and major advances have been made to decrease postoperative morbidity and mortality [2, 3]. These improvements in perioperative care have allowed for the emergence of other endpoints to assess improvement in perioperative care [4]. Patient-rated quality of recovery [5, 6] after surgery is gaining importance as a relevant endpoint in the study of perioperative care.

Application of pressure to specific external sites on the body has been practiced for hundreds of years and is generally recognized as a safe, cost-effective, non-invasive form of therapy with few to zero adverse effects [7, 8]. A meta-analysis studying the effectiveness of PC6 acupoint stimulation versus sham treatment or antiemetic drugs for the prevention of postoperative nausea and vomiting (PONV) including 59 trials [9] found a beneficial effect for PC6 acupoint stimulation versus sham treatment and no differences for PC6 acupoint stimulation versus antiemetic. This meta-analysis recommended further high-quality research on PC6 acupoint stimulation and on other acupoints. For pain treatment, a randomized controlled trial with a sample size of 129 patients [10] comparing acupressure and physical therapy for low back pain treatment showed a beneficial effect on pain scores in the acupressure group. A randomized controlled trial by Chen et al. [11] comparing acupressure versus no acupressure on sleep time and quality for intensive care unit patients (*n* = 85) showed beneficial effects in the acupressure group. The studied acupoints by Chen [11] were Neiguan (PC6), Shenmen (HT7) and Yongquan (K11) [12]. Anxiety improvement is also a studied field for acupressure therapy, and Beikmoradi et al. [13] showed that acupressure therapy including ear cavity, LI4, LI10, HT7, LU9, DU20, Ren6, Yintang and UB13 was beneficial compared to sham acupressure and control (*n* = 90).

Therefore, several studies suggest that acupressure therapy may be potentially beneficial in improving postoperative symptoms like PONV, pain and sleep disorder and improving postoperative quality of recovery. However, high-quality evidence to show the beneficial effects of acupressure therapy in postoperative quality of recovery care is lacking. For example, in a Cochrane meta-analysis on PONV [9], the primary outcome analysis on PC6 acupoint stimulation and antiemetic combination versus antiemetic alone was graded as very low quality due to heterogeneity, limitations and imprecision among the trials.

This three-group, parallel, superiority, blinded, randomized controlled trial will test the hypothesis that a combination of PC6, LI4 and HT7 acupressure is superior to sham treatment or no intervention for improving postoperative quality of recovery in hospitalized patients for surgical treatment. This therapy, if efficient, could help in improving quality of recovery following a large group of surgical procedures with few potential side effects.

## Methods/design

### Study design

**Fig. 1:**
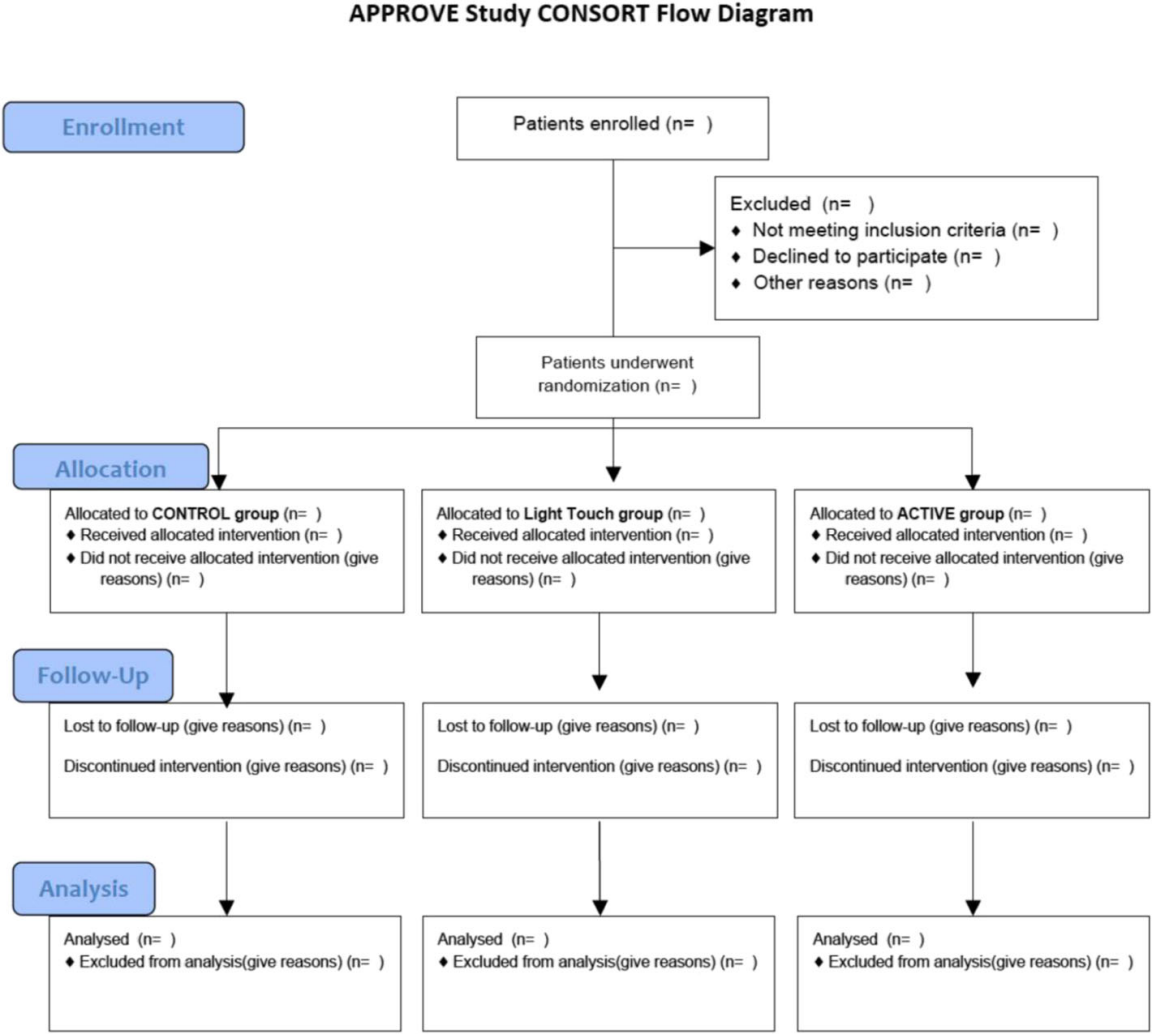
The Consolidated Standards of Reporting Trials (CONSORT) flow diagram template for the AcuPressure to improve Patient satisfaction and quality of Recovery (APPROVE) study

The study is a three-group, parallel, superiority, randomized controlled trial (Fig. 1). Participants will be postoperative adult inpatients. The tested intervention will be PC6, LI4 and HT7 acupressure therapy. The main outcome will be quality of recovery with secondary outcome assessments of patient satisfaction, PONV and pain by the third postoperative day. The use of two comparator groups, i.e. a sham (light touch) group and a no intervention group (control group), will allow the assessment of different aspects of the placebo effect, including behavioural aspects (visiting and interacting with the patient) [14]. Research team members obtaining survey and other outcome data will not be told the allocated group identities. The study design aims at fulfilling the Standard Protocol Items: Recommendations for Interventional Trials [15] (Additional file 1: SPIRIT 2013 checklist) and the Revised Standards for Reporting Interventions in Clinical Trials of Acupuncture (STRICTA): extending the CONSORT Statement [16].

### Setting and population

After Institutional Review Board approval, ClinicalTrials.gov registration and written informed consent, adult study patients will be enrolled. One hospital site will be involved in the study reflecting homogenous perioperative care. Only surgical patients expected to stay at least 2 days at the hospital after surgery and able to answer the study questions will be included. Recruiting time is planned to take 4 months.

### Sample size

Sample size calculations were carried out using SAS© 9.4 software, SAS, Cary, NC, USA. Based on results from a study examining the psychometric properties of the quality of recovery (QoR-15) evaluation [6, 17], we estimate the overall change from baseline to follow-up in untreated patients will be approximately 5% of the instrument total, or +7.5 points. We powered the study to be able to detect a clinically relevant change of 10% (+15 points) in the treatment group, with a standard deviation of 12 points. The primary hypothesis is a better quality of recovery in patients randomized to the active acupressure intervention group. A sample size of 50 subjects per arm (150 total) was calculated based on a two-sample *t* test for mean difference, with power = 0.85, alpha = 0.05. We have requested a total of 200 patients consented in order to allow for some screen failures that will inevitably occur, e.g. cancelled surgery.

### Recruitment

Patients will be screened at an academic center, the Stony Brook Medicine Hospital (Stony Brook, NY, USA) by a research team member. Those meeting all the inclusion criteria and none of the exclusion criteria will be introduced to the study. If interested, patients will be provided with complete information about the study including explanation, information sheet and clarification of any questions. Contact information for future questions will be provided and written informed consent recorded.

Inclusion criteria are English-speaking patients at Stony Brook Medicine Hospital who are expected to stay in the hospital for at least 2 days and are able to provide written informed consent.

Exclusion criteria are as follows: patients younger than 18 years of age; those who do not have access to the three points on the hand and wrist due to skin breakdown, ulcers, cellulitis, broken bone, indwelling catheter within 5 cm radius of the pressure points, etc. (since the plan is to apply the pressure unilaterally, this exclusion only applies if the issue that is preventing pressure to be applied exists on both extremities); significant dementia or altered mental status that would prevent assessment of the QoR-15 survey; allergic reaction to ink from a Sharpie pen; stroke or other neurologic condition which precludes sensation in both upper extremities; use of regional anaesthetic technique, e.g. epidural, continuous peripheral nerve catheter, after postoperative day 1 at 12 noon; women who are currently pregnant, screened via urine pregnancy test.

### Randomization and allocation concealment

**Fig. 2:**
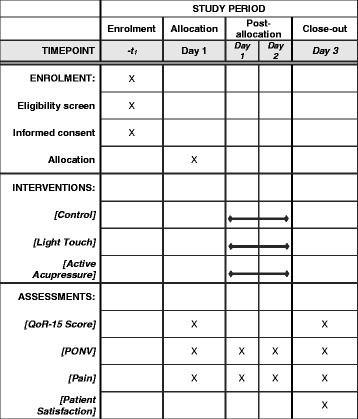
APPROVE study schedule of enrolment, interventions and assessments

Randomization will be performed using a sealed envelope technique. Envelopes will not be reused in any case. Patients will be randomly allocated to one of the three experimental groups (Figs. 1 and 2): control (no visit), light touch (sham acupressure) or active acupressure therapy. A 1:1:1 ratio computer-generated randomization schema stratified on the use of postoperative regional anaesthesia in random permuted blocks of varying sizes will be provided by the trial’s statistician. To keep the statistician blinded to the study groups, the statistician will provide the randomization schemes in an A/B/C format; an independent individual not otherwise involved in the study will finalize the assignment of the actual treatment arm and prepare the sealed envelopes. Each participant will be randomized (i.e. envelope opened) before the first intervention but after marking of the three acupoints with a Sharpie pen by research team members (including SS, CM, EN).

### Process to ensure blinding

Patients, routine care providers and team members performing outcome assessment visits will not be told of the patients’ study group allocation. Allocation will only be revealed on request from routine care providers if necessary to change the management of the patient. All other team members (including SS, CM, EN) will apply the active/light touch interventions. Study acupoints will be marked on all patients to ensure assessor blinding.

### Intervention

Acupoints will be marked on every patient before randomization using a Sharpie pen. As recommended by experts [18], the first choice side is left for males and right for females

**Fig. 3:**
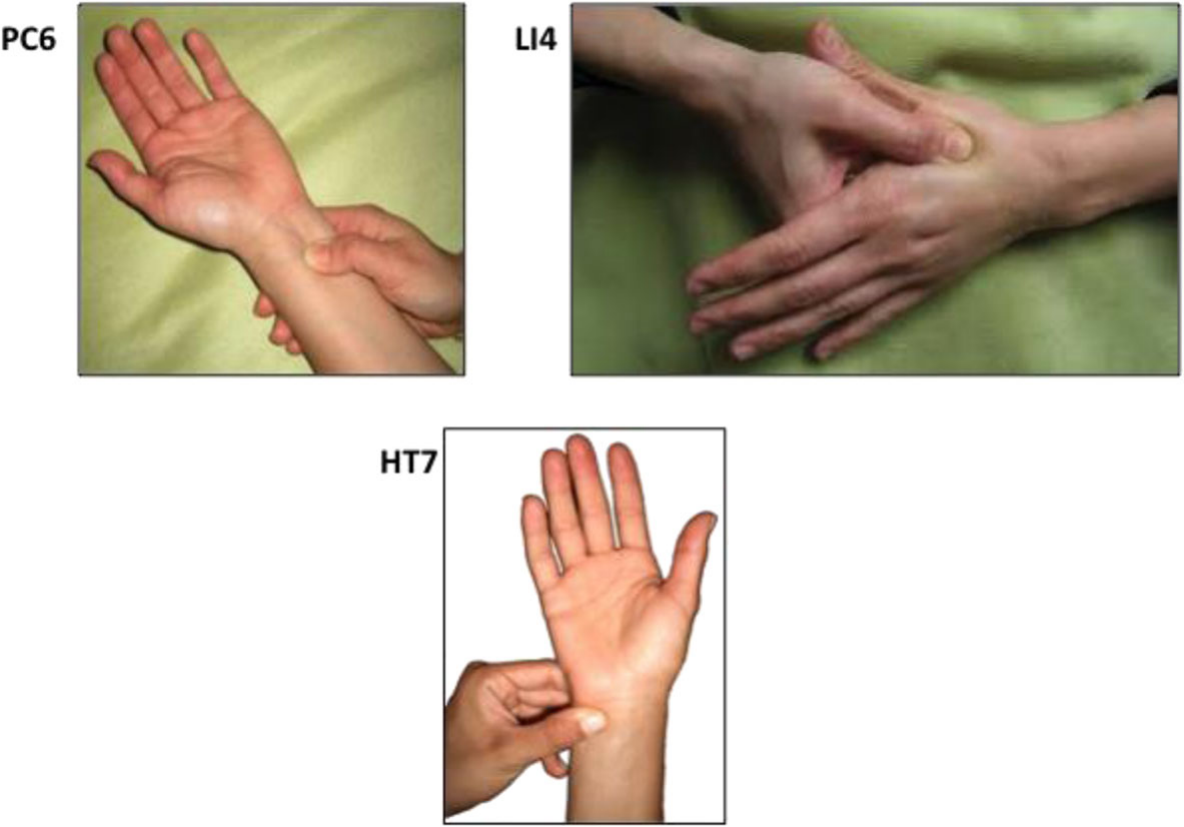
PC6, LI4 and HT7 acupoint locations. The acupoints are represented by the applied thumb position

*In the active intervention group*, acupressure therapy will consist of unilateral thumb pressure application on the following acupoints [12]: PC6 for 2 minutes, then approximately 1 minute rest, then LI4 for 2 minutes, then approximately 1 minute rest, and then HT7 for 2 minutes. PC6 is two patient interphalangeal thumbs width proximal to the anterior wrist crease between the flexor carpi radialis and palmaris longus, LI4 is at the center between the 1^st^ and 2^nd^ metacarpal bones and HT7 anterior wrist crease, proximal to the pisiform and lateral to the flexor carpi ulnaris tendon [12] (Fig. 3). Acupressure involves a very strong level of pressure, which is often just below the level that elicits pain, whereas the light touch group is merely touched, with no significant pressure involved. Research team members performing interventions will be trained to perform a standardized pressure defined by an expert acupressure therapist (XG) using high frequency feedback simulation. High frequency feedback consists of a training plan based on practicing to apply pressure on a weighting scale to mimic the expert pressure values. The trainees were given feedback on the applied pressure at high frequency (every minute). In our training program for study personnel we used a scale to show how much pressure should be used for acupressure (approximately 5000 g) vs. light touch (approximately 50 g), which represents a 100-fold difference in level of pressure. Using weighting scale-based simulation training for pressure application has already been validated for cricoid pressure training [19].

*In the light touch group*, intervention will consist of the thumb lightly touching the same acupoints, during the same time and in the same order as the acupressure group. Research team members will also be trained to perform standardized light touch defined by an expert acupressure therapist (XG) using high frequency feedback simulation. Both the acupressure and light touch procedures will follow the same standardized procedural protocol.

The interventions (acupressure or light touch) will start at the first postoperative day in the morning after randomization has occurred (day 0) and end on study day 2 in the evening. Interventions will be performed three times a day: between 7:00 to 10:00 am, 12 noon to 2:00 pm and 5:00 to 7:00 pm.

*In the control group*, no visit will be performed except for the endpoints assessment.

Interventions will be terminated only on patient request or if an exclusion criterion later occurs.

### Concurrent treatment

All patients will receive routine care. To assess balance between the three groups in potential confounders, variables such as age, sex, comorbidities, type of surgery, type of anaesthesia, type of anaesthetic agents, prophylactic antiemetics and sleep aids will be recorded. Clinicians involved in routine patient care will be blinded to study group assignment.

### Outcome measures

Data will be collected using an electronic case report form and a few written forms created to maintain blinding of the assessors. The primary endpoint will be the change in the established quality of recovery (QoR)-15 score [6]. The QoR-15 is a 15-question survey measuring patient satisfaction/quality of recovery including pain, nausea, sleep and well-being. Each question is rated on a Likert scale from 0 to 10. The maximum score of the QoR-15 is 150 points, indicating ideal health status. This score will be assessed by a research team member on study day 0 between 7:30 and 8:30 am prior to first intervention and on study day 3 between 7:00 and 10:00 am. If the patient is discharged from the hospital before study day 3, the QoR-15 score will be completed by telephone.

Secondary endpoints include individual measures of pain, nausea and patient satisfaction, using a numeric rating scale, and vomiting that will be assessed on study days 0 (between 7:30 and 8:30 am, i.e. before the first intervention), 1 and 2 (between 3:00 and 4:00 pm). Patient opinion about acupressure effect will be assessed on study day 0 between 7:30 and 8:30 am prior to the first intervention and on study day 2 between 7:00 and 10:00 am.

Other secondary endpoints will be recorded daily from the patient medical record, including sleep aids, antiemetic administration, opioid and non-opioid consumption, episodes of delirium and hospital length of stay. A research coordinator will monitor accuracy of the data in randomly sampled case report forms by comparing hospital source documents with data in the electronic case report form. QoR-15 scores will be recorded on paper case report forms and later entered into an MS Access© database. Study documents will be stored in a locked cabinet. Study team members will have access to the final trial dataset.

### Data analysis

#### Analysis plan

This is a randomized, single-blinded controlled trial. The study will be analysed in four ways. The primary method of analysis will use the modified intention to treat (mITT) approach, with the treatment group incurring at least one treatment session. Patients who obtain the baseline measurement and are randomized to the treatment group but do not receive at least one treatment will be excluded from the analysis. Otherwise, missing data will be accounted for using a multiple imputations method [20]. In addition, if data are missing at random, a secondary sensitivity analysis will be conducted that excludes patients with missing follow-up data, but includes patients who did not complete the entire treatment regimen (follow-up complete, FC). A tertiary adherers-only (AO) analysis may be included to examine the subset of patients who fully received the treatment to which they were randomized and completed both baseline and follow-up assessments. We will also perform an ITT analysis.

#### Statistical analysis

The primary outcome of this study is the QoR-15 change score from baseline to follow-up on day 3. Mean change score between the control group (no intervention) and the acupressure treatment group will be assessed for differences using either the Student’s *t* test or Wilcoxon rank sum test. We do not expect to see any significant differences between the two groups regarding age, sex, American Society of Anesthesiologists (ASA) status, surgical duration, anaesthetic technique, intraoperative fluid therapy volume, postoperative day 1 Sequential Organ Failure Assessment Score [21] and other comorbidities due to the randomization process; however, we will examine the groups for differences. Additional event metrics (e.g. antiemetic medication consumption and opioid consumption (including amount)) during the study period will also be collected and analysed. Event variables that differ significantly between groups may be used as potential covariates in a multivariate linear regression. The statistician will remain blinded to the group assignment for the analyses.

Finally, for all four methods of analysis (mITT, FC, AO, ITT), secondary assessments for the placebo effect will be conducted. Analyses for differences in mean change QoR-15 score include comparisons between (1) the control vs. sham group and (2) the sham group vs. the active acupressure group.

## Discussion

### Acupressure intervention

We selected three acupoint locations, based on recommendations by the World Health Organisation [12]. These points are commonly used for treating symptoms that may improve quality of recovery like PONV, pain and sleep disturbance. These acupoint locations will be marked accordingly on every patient before randomization to improve the standardization of the intervention and decrease the hazard of measurement bias.

Multipoint acupressure [22–25] was preferred over single point acupressure to increase the likelihood of triggering a beneficial signal. The combination of multiple acupressure points was shown to be beneficial for ventilator-induced anxiety and dyspnoea [22], for prehospital analgesia in patients with radial fractures [25] and for prehospital analgesia in patients with minor trauma [26].

We chose to apply acupressure for 2 minutes on each point at each session. This duration has already been used in published randomized controlled trials reporting beneficial effects of acupressure therapy. Wang and colleagues reported beneficial effects on self-assessed quality of life for hemodialysed patients [27] using 2 minutes of pressure on auricular acupoints. Similary, Pouresmail described positive effects of 2 minutes of acupressure on primary dysmenorrhea [28]. Yang et al. [29] reported favourable effects of 2 minutes per point acupressure associated with aromatherapy on dementia-associated agitation.

The frequency of the intervention was set as three times a day, similar to previously published studies, including those of Chang et al. for postoperative pain after total knee replacement [30] and Shin et al. for hyperemesis gravidarum [31].

The treatment will be administered for 2 days, which was a balance between more sessions (i.e. 6 days) that might confer more benefit with a need to limit the sessions to a period of time when these hospitalized patients would still be available for the study interventions.

### Outcome measurements

This trial aims at assessing the effectiveness of acupressure therapy on quality of recovery and patient satisfaction in hospitalized patients. The QoR-15 score was developed to determine a self-rated patient measure of overall health status [6] and has undergone psychometric and external validation [17]. The QoR-15 score is easy to administer and has been validated to detect clinically important differences in patient-perceived health status [32]. Therefore, we believe it is a relevant endpoint for assessing acupressure therapy effectiveness in this setting.

### Control groups

Control groups are very challenging to define when studying complex non-pharmacological therapies like acupressure. Control group treatments may consist of standard care without any additional intervention, moderate pressure on a sham acupoint or light touch on a real acupoint. Each of these controls may explore another aspect of the placebo effect being part of the acupressure therapy [14]. In this study we choose two control groups: no intervention at all and light touch on the real acupoints (type 3 sham methods according to the Tan classification [14]). This will allow us to explore the contribution of the placebo effect induced by direct human contact and interaction in the light touch group.

### Trial Status

Screening began on 28 March 2016.

## Additional file

Additional file 1: SPIRIT 2013 checklist. (DOC 120 kb)
